# Ecology of prokaryotic DNA viruses in a highly impacted coastal lagoon revealed through comparative and temporal metagenomics

**DOI:** 10.1093/ismeco/ycag110

**Published:** 2026-04-21

**Authors:** Guillermo Domínguez-Huerta, Ana María Cabello, Jorge J Santos-Bruña, Jesús M Mercado, Isabel Ferrera

**Affiliations:** Centro Oceanográfico de Málaga, Instituto Español de Oceanografía, IEO-CSIC, Málaga 29002, Andalusia, Spain; Centro Oceanográfico de Málaga, Instituto Español de Oceanografía, IEO-CSIC, Málaga 29002, Andalusia, Spain; Centro Oceanográfico de Málaga, Instituto Español de Oceanografía, IEO-CSIC, Málaga 29002, Andalusia, Spain; Centro Oceanográfico de Málaga, Instituto Español de Oceanografía, IEO-CSIC, Málaga 29002, Andalusia, Spain; Centro Oceanográfico de Málaga, Instituto Español de Oceanografía, IEO-CSIC, Málaga 29002, Andalusia, Spain

**Keywords:** viral ecology, prokaryotic viruses, metagenomics, coastal lagoon, Mar Menor, eutrophication

## Abstract

Coastal lagoons are highly productive ecosystems, yet their prokaryotic viruses remain poorly studied. The Mar Menor, a hypersaline coastal lagoon in south-eastern Spain, is under strong anthropogenic pressure from continuous agricultural runoff, leading to severe eutrophication. To assess the impact of these unique conditions on viral assemblages, we analyzed a 3-year metagenomic time series of picoplankton communities. We reconstructed the lagoon’s prokaryotic DNA viral communities and compared them with their counterparts in open Mediterranean Sea waters to reveal how environmental variability shapes their structure. Viral communities in the Mar Menor showed higher viral operational taxonomic unit relative abundances and diversities and were distinct from those offshore. Temporally, community structure was correlated with water transparency and silicate concentration. The putative hosts of the lagoon viruses were copiotrophic rather than oligotrophic compared to the open sea, and their composition shifted in response to episodic environmental disturbances. Temperate virus levels did not consistently support either the piggyback-the-winner or refugium models, spatially or temporally, indicating that viral replication strategies are governed by factors more complex than trophic status or environmental variability alone. Auxiliary viral genes (AVGs) encoding 2-oxoglutarate/Fe(II)-dependent oxygenase and DNA methyltransferase emerged as potentially relevant functions in the lagoon, as they were more frequent than in the Mediterranean Sea. Similar to targeted hosts, AVG-specific temporal relative abundance patterns were strongly shaped by local disturbances. This study provides the first metagenomic insight into viruses of the Mar Menor, revealing viral ecology in a dynamic, eutrophic lagoon, with implications for predictive models of nutrient cycling.

## Introduction

Coastal lagoons are shallow water bodies with restricted exchange with the open sea. They are influenced by both marine and freshwater inputs, making them dynamic and highly productive systems [1] that offer important ecosystem services, such as carbon sequestration and fisheries [2]. However, these ecosystems are particularly vulnerable to anthropogenic pressures, with eutrophication being a primary impact. This process, driven by high-nutrient loads, often leads to algal blooms and hypoxia, threatening the ecosystem integrity [3].

Despite the ecological significance of coastal lagoons, studies of their microbiomes remain scarce [4], particularly on the viral component, even though viruses are nowadays recognized as key players of marine food webs and biogeochemical cycling [5]. Existing studies have primarily focused on human and fish pathogens [6–10], rather than investigating the ecology of planktonic viruses [11–15]. Due to their shallow depth, restricted water exchange, and strong land-sea coupling, coastal lagoons are susceptible to perturbations and exhibit strong natural environmental variability. Rather than obscuring long-term signals, this variability amplifies ecosystem responses, allowing climate- and pollution-driven effects to emerge more rapidly and intensively than in buffered open marine systems. Consequently, coastal lagoons represent valuable natural laboratories to investigate the impacts of climate change (e.g. marine heatwaves) and anthropic pollution (e.g. eutrophication) on viral communities [16–18], which is crucial for predicting the marine microbiome responses to global challenges [19, 20]. Additionally, they provide a natural scenario to study a central topic in viral ecology: the lysis–lysogeny switch under conditions of high nutrient concentration (i.e. active host growth) [21–24] and strong environmental fluctuations (i.e. amplitude of host physiological changes) [12–14]. Nevertheless, the host range and metabolic reprogramming of viruses in coastal lagoons remain unexplored, limiting our understanding of their ecological footprint.

This study leverages the unique environmental characteristics of the Mar Menor, a coastal lagoon in south-eastern Spain that was once Europe’s largest hypersaline lagoon. Historically an oligotrophic system, the Mar Menor has experienced significant nutrient loading (mainly nitrates) from intensive agriculture since the 1990s [25], which prompted a severe eutrophication event in 2016, leading to recurrent phytoplankton blooms and fish mortality episodes over the past decade. High-throughput sequencing was applied for the first time to study the bacterioplankton of the Mar Menor in 2010, before the eutrophic crisis, reporting dominant sulfur-oxidizing alphaproteobacteria and *Synechococcus* [11]. Based on optical microscopy, the eukaryotic phytoplankton was characterized in 2016–2021, determining that the trophic status of the lagoon has changed profoundly [26]. More recently, 16S rRNA gene and metagenome sequencing revealed that the bacterioplankton community respond rapidly to short-term environmental fluctuations [27, 28]. Nevertheless, a comprehensive viral metagenomic study in the Mar Menor is still lacking.

Here, we analyzed picoplankton metagenomes (0.2–3 μm size fraction) collected between October 2019 and October 2022 in the Mar Menor to characterize prokaryotic DNA viral communities, including normalized relative abundance of their viral operational taxonomic units (vOTUs) (via metagenomic read mapping), diversity, community structure, hosts, replicative strategies, and reprogramming of host metabolism and physiology. Our main objectives were (i) to understand the impact of eutrophication on the composition of viral communities and (ii) to explore the influence of temporal environmental gradients and episodic perturbations in the lagoon. To accomplish these objectives, we employed two complementary approaches: (i) compared the viral communities of the Mar Menor to communities from various oligotrophic locations across the open Mediterranean Sea and (ii) analyzed their seasonal and interannual variability. We hypothesize that viral communities in the Mar Menor show distinct ecological signatures compared to those in the open Mediterranean Sea, due to their intrinsic dependence on aquatic microbiomes (and the expected correspondence between intracellular viruses in metagenomes and prokaryotic community dynamics [29]), which have been strongly impacted by the eutrophication and environmental shifts experienced by the lagoon [26, 27]. Alternatively, hydrological connectivity with the open sea could override local selection pressures, making the viral communities indistinguishable. Overall, our findings highlight the importance of investigating viruses in aquatic systems subjected to excessive nutrient inputs and environmental disturbances, as these pressures are increasingly frequent and profoundly reshape microbial dynamics.

## Materials and methods

### Sample and environmental data collection

The Mar Menor water samples and ancillary data were obtained as part of a time-series monitoring program run since 2016 by the Spanish Institute of Oceanography. Our metagenomic sampling took place in one station located in the center of the lagoon (Supplementary Fig. 1) and covered a 3-year period, from October 2019 to October 2022 (Supplementary Fig. 2A). Acquisition methods of the lagoon’s environmental data were described elsewhere [30]. Water samples and environmental variables [temperature, salinity, fraction of incident photosynthetically active radiation reaching the bottom of the water column (I_z_%) (used as a reference to quantify light attenuation or water transparency), dissolved oxygen, the concentrations of nitrite, nitrate, phosphate, silicate, total nitrogen, total phosphorous, chlorophyll *a*, and cell abundances of picoeukaryotes, heterotrophic prokaryotes, and *Synechococcus*] (Supplementary Fig. 2B) were obtained from samples collected at a depth of ~4 m.

To concentrate picoplankton biomass, ~0.6–1 l of seawater, prescreened by a 200 μm mesh, was sequentially filtered through 3 μm and 0.2 μm pore-size polycarbonate membrane filters (47 mm diameter, DHI) using a peristaltic pump (Supplementary Fig. 3). Filters were stored at −80°C until DNA extraction. Filters were cut into pieces using sterile razor blades before being processed employing the DNeasy PowerSoil Pro Kit (QIAGEN) following the manufacturer’s instructions. The quality and the concentration of the extracted DNA were evaluated by spectrophotometry using a BioDrop Touch Duo and fluorometry utilizing the Qubit technology (Life Technologies), respectively. The extracted DNA was stored at −80°C until shotgun metagenomic sequencing was performed at Novogene, UK (www.novogene.com). Libraries were prepared using the kit Novogene NGS DNA Library Prep Set (Cat No.PT004). The fragments with adapters were size-selected to 350 bp, PCR amplified, and purified. The libraries were checked with Qubit and real-time PCR for quantification and a Bioanalyzer for size distribution detection. Quantified libraries were pooled and sequenced on the Illumina platform NovaSeq6000, and paired-end reads were generated (strategy PE150).

### Retrieval of previously published metagenomes for comparative analysis

For the Mediterranean picoplankton metagenomes, we downloaded the Illumina-generated sequencing data from the Sequencing Read Archive (SRA) database. To perform comparative analytics with picoplankton samples from the Mediterranean Sea, we selected metagenomes from two sources: 0.22–5-μm size fractions from the vicinity of the Mar Menor (~200 l) [29] (one metagenome) and 0.22–1.6-μm size fractions from the *Tara* Oceans expedition (~100 l) [31] (six metagenomes; Algerian basin, Menorca-Sardinia channel, Sicily channel, Adriatic Sea, Ionian Sea, and Levantine basin). All sequencing data were processed using the bioinformatic pipeline described below. We also downloaded metagenomic pyrosequencing data for the Mar Menor and the Albufera of Valencia [11] from the Integrated Microbial Genomes & Microbiomes system (IMG/M) [32]. As reported in the original publication [11], water samples from the Albufera de Valencia (~20 l) and the Mar Menor (~40 l) were sequentially filtered using three different filters (0.1 μm, 0.8 μm, and 3 μm), and the extracted DNA was sequenced with the Roche 454 GS-FLX system. For our cross-habitat comparisons, we utilized the assembled contigs obtained by pooling the three plankton sizes for both locations, which were then processed through our viral identification pipeline.

### Metagenome assembly and identification of viral contigs

Raw metagenomic reads were trimmed with TRIMMOMATIC v0.39 [33] using the following parameters to remove low-quality reads: LEADING:3 TRAILING:3 SLIDINGWINDOW:4:15 MINLEN:50. Assembly of paired reads was performed using MEGAHIT v1.2.9 [34] with the meta-large preset. Contig sequences larger than 5 kb were searched for prokaryotic double-stranded and single-stranded DNA viruses, following the standard operating procedure recommended by Guo *et al.* [35]. As we sampled picoplankton DNA, bioinformatic screening of their metagenomes targeted mostly intracellular viruses, either in lytic or lysogenic cycle. Briefly, a first VirSorter2 [36] pass was applied using a score of 0.5, and candidate viral contigs were later inspected and sorted into confidence categories using CheckV [37]. The latter software was also employed to estimate viral genome completeness and remove host regions from proviruses. Functional annotation with DRAMv [38] was utilized to manually inspect contigs carrying suspicious cellular genes that are usually present in both viruses and prokaryotes.

### Clustering into viral operational taxonomic units and calculation of their normalized relative abundances

The observed viral contig sequences were clustered into vOTUs using BLASTn-based software and the scripts available on the CheckV bitbucket repository [39], following previously recommended thresholds of 95% of average nucleotide identity and 80% of length coverage of the shortest contig [40]. This clustering approach has been previously proposed to yield vOTUs, representing populations *sensu* Brum *et al.* [41].

Quality-trimmed reads were mapped to the representative contigs using CoverM v.0.7.0 [42], requiring ≥95% of nucleotide identity, ≥75% read alignment, and ≥70% contig coverage. The trimmed mean of the number of reads that mapped to a nucleotide position was divided by the total number of nucleotides in all the quality-trimmed reads of the corresponding sample and multiplied by 10^9^ to normalize by metagenome gigabase.

### Characterization of the viral operational taxonomic units

To assign viral taxonomy, representative contig sequences whose lengths were ≥10 kb were used as input for vConTACT3 [43], a software based on the analysis of gene-sharing network, with the ProkaryoticViralRefSeq211-Merged (25 April 2022), using default parameters. The putative host genera of the representative contig sequences of the vOTUs were predicted using iPHoP (v1.3.3) [44], retaining only predictions with scores ≥90. The replicative strategy (virulent and temperate) was predicted for vOTUs with complete or high-quality genomes using the software BACPHLIP v0.9.3-alpha [45] with probability ≥95%. To predict temperate viruses beyond high-quality genomes, we built Hidden Markov Models (HMMs) from previous alignments of viral recombinases [46] using the program hmmbuild of HMMER v3.3.2. The protein sequences predicted through DRAM-v were searched for these HMMs using the program hmmsearch with bit score ≥50. The annotation of lower-score hits was confirmed with the CD-Search tool of NCBI Conserved Domain Database v3.21 [47]. The “temperate virus” prediction was assigned to the entire vOTUs, as they putatively belong to the same population. All virus contigs were first annotated using DRAM-v (v 1.4.6) [38] with a bit score ≥ 60 to assign database identifiers to gene. Then a “distill” step was utilized to retain those auxiliary viral genes (AVGs) with an auxiliary score ≤3 and without the V amg_flag. To ensure conservative annotation, we transferred the functional annotations of the Kyoto Encyclopedia of Genes and Genomes (KEGG) from the annotations.tsv to these AVGs. Functional categories were assigned based on searching the KEGG-based annotations in the release 2025_03 of UniProt [48]. AVGs were assigned to the corresponding vOTUs, as they putatively belong to the same population. To find a specific function for the 2-oxoglutarate (2OG)/Fe(II)-dependent oxygenases, we employed two strategies. First, we retrieved 57 reviewed bacterial protein sequences annotated with the EC 1.14.11 from the UniProt Knowledgebase (UniProtKB; as of 24 September 2025) and pooled with the 2OG/Fe(II)-dependent oxygenases encoded by our vOTUs. The combined set was used to build a database with BLAST+ [49], which was subsequently employed to construct a sequence-similarity network with an e-value threshold of 10^−5^. The network was cleaned of self and reciprocal matches and visualized using Cytoscape v3.10.3 [50]. Second, to identify specific function domains, the protein sequences from both the UniProtKB and viral 2OG/Fe(II)-dependent oxygenases were searched against the NCBI Conserved Domain Database [47] using the Batch CD-search tool with an e-value threshold of 10^−2^.

### Data visualization and statistical analyses

All calculations, statistics, and plots were generated in R (v4.3.2) [51]. Maps were created using Ocean Data View (https://epic.awi.de/id/eprint/56921/). Alpha-diversity was computed with the vegan package [52], and richness was additionally normalized per gigabase of quality-checked reads. For time-series analyses, centered log ratio (CLR) transformation was applied to normalized relative abundances of vOTUs due to a zero-inflated distribution, previous normalization was applied with a pseudo-count of 0.001. To identify significant differences between these normalized relative abundances, nonparametric Friedman test was applied because no normal data was diagnosed with the Anderson–Darling test (nortest). Following, when differences were detected, two-sided Wilcoxon signed-rank tests with false discovery rate (FDR) correction were applied to determine contrast between groups. Beta-diversity was evaluated using Aitchison distances applied to normalized relative abundances of vOTUs (Euclidean on CLR-transformed data with compositions), visualized by principal coordinate analysis (PCoA) with stats::cmdscale and clustered by *k*-means; *k* was chosen as the lowest value yielding the highest silhouette width with cluster. Environmental drivers were fitted to ordinations with vegan::envfit (999 permutations). Group differences were tested by PERMANOVA (adonis2) with pairwise comparisons (pairwiseAdonis) and FDR correction. Overlap of vOTU used the Sørensen–Dice coefficient [100 × Shared/mean(*n*_1_,*n*_2_)] [53]. Temporal distance-decay was assessed from Bray–Curtis dissimilarities (log[*x* + 1]) and partitioned into turnover and abundance-gradient components (betapart), with Mantel tests. Between-group differences (Mar Menor vs. open Mediterranean Sea) regarding the analyzed viral features were tested using two-sided Wilcoxon rank-sum tests with FDR correction. Effect size calculations on vOTU percentages (regarding hosts, replicative strategy, and AVG functions) included zeros and retained only cases with ≥5 vOTUs in ≥1 group and median differences ≥0.1. In addition, because replicative strategy and AVG annotations derive from contigs in clusters, group-shared vOTUs were excluded. Heatmaps and hierarchical clustering used pheatmap on log10[*x* + 1] values. Portions of the R scripts used in this study were reviewed with the assistance of artificial intelligence using ChatGPT (OpenAI).

## Results and discussion

### Mar Menor viral communities showed higher viral operational taxonomic unit relative abundance and diversities, and are compositionally distinct

We identified a total of 31 647 viral contigs in our 14 Mar Menor samples (Supplementary Dataset 1). Moreover, following the same pipeline, we identified 10 711 viral contigs in 6 publicly available picoplankton metagenomes generated with comparable Illumina sequencing, across different regions of the open Mediterranean Sea (see Materials and methods; Supplementary Fig. 1). The lagoon viral contigs were clustered into 22 534 vOTUs. When clustered together with the Mediterranean viral contigs, we obtained 30 637 vOTUs, which were utilized to compare diverse viral features across the two ecosystems. Despite methodological differences between the datasets, the combined data still support a robust comparison that reveals clear biological contrasts (discussed in detail in Supplementary Information).

**Figure 1:**
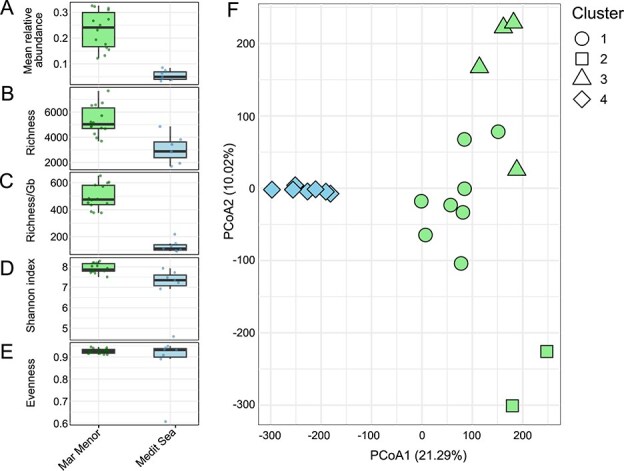
Comparative analysis of viral communities across the Mar Menor and the Mediterranean Sea. Boxplots show mean relative abundance per sample of (A), observed richness (B), richness normalized per gigabase of metagenomic reads (C), Shannon diversity (D), and Pielou’s evenness (E) highlighting differences between the two ecosystems. The structure of the communities from both ecosystems was compared with PCoA using a fixed number of four clusters that were assessed with *k*-means clustering analysis (F). Percentages on the axes indicate the proportion of total variance explained by each PCoA dimension.

Both vOTU relative abundance and alpha-diversity were consistently higher in the lagoon than in the open Mediterranean Sea (Wilcoxon test, *P* < .05) as indicated by the mean vOTU relative abundance (4.8-times, *P* = .000), richness (1.75 times, *P* = .002), richness normalized by metagenome size (4.27 times, *P* = .0003), and Shannon diversity (1.07 times, *P* = .004) (Fig. 1A–D, Supplementary Dataset 1). The lack of a significant difference for evenness (Fig. 1E, *P* = .322) suggests that the observed higher Shannon diversity is caused by the higher number of vOTUs in the Mar Menor. Such higher diversity was further supported by the occurrence of lagoon-exclusive viral taxa (*Zobellviridae, Crassvirales, Tokiviricetes*, and *Malgrandaviricetes*) (Supplementary Dataset 1). Our results suggest that the eutrophic conditions in the lagoon sustain both high vOTU relative abundance and diversity, consistent with observations in trophic gradients [54–56]. These patterns are likely associated with different mechanisms previously proposed in the literature [16, 57] including (i) higher host cell density, (ii) higher levels of host activity (as experimentally demonstrated [58–60]), and (iii) switch from lysogeny to lysis [16]. In our analyses, it was not possible to determine the relative contribution of prophages to vOTU relative abundance, as the fragmented Illumina-derived contigs hamper our capability to detect provirus-host boundaries [61]. Additionally, beyond host physiology, other features of the lagoon, such as frequent environmental fluctuations and limited dispersal typical of a semi-enclosed system, may also contribute to the elevated viral alpha-diversity by supporting a cumulative and broader host community. In conclusion, we discard the hypothesis that the hydrological connectivity of the lagoon with the open sea overrides local selection pressures.

In addition, we investigated the compositional similarity between Mar Menor viral communities and those from the open Mediterranean Sea (Supplementary Fig. 1). PCoA (Fig. 1F) explained 31.3% of the total variance and revealed a clear separation between lagoon and open-sea viral communities. Concordantly, *k*-means analysis permitted identifying four sample clusters (*k* = 4; Supplementary Fig. 4A) whose differences were assessed by a PERMANOVA test (*r*^2^ = 0.39, *P* = .001; Supplementary Dataset 2). Post hoc tests also supported statistically significant differences among clusters 1 vs. 3, 1 vs. 4, and 3 vs. 4, while comparisons involving cluster 2 were less robust. These results suggest pronounced environmental divergence despite connectivity between the water masses of the Mar Menor and the open Mediterranean Sea. Consistently, the percent of shared vOTUs between the Mar Menor and diverse regions of the open Mediterranean Sea was very low (0.0%–1.2%), contrary to the higher overlap observed among the open-sea regions themselves (2.2%–30.0%) (Supplementary Fig. 5A, Supplementary Dataset 2). The open-sea communities with the highest overlap of vOTUs with the Mar Menor were in the Ionian Sea (0.1%–1.2%), the Algerian basin (0.1%–0.7%), and the open-sea region closest to the Mar Menor (near-MM, 0.0%–0.6%) regions, which possibly points to an effect from geographical proximity (near-MM and Algerian basin).

Additionally, we examined the level of shared vOTUs with previously pyrosequenced metagenomes from samples collected in 2010 from both the Mar Menor and the Albufera of Valencia, a eutrophic freshwater coastal lagoon in eastern Spain, about 250 km north of the Mar Menor [11] (see Materials and methods) (Supplementary Fig. 5A; Supplementary Dataset 2). Notably, there was a very low level of shared vOTUs when comparing the pyrosequenced metagenome from the Mar Menor with Illumina-sequenced metagenomes from either the Mar Menor time series (0.1%–0.9%) or the open Mediterranean Sea (0.0%–0.4%). Despite the intrinsically lower sequencing depth of pyrosequencing compared to Illumina, this observation might still reflect the different environmental lagoon conditions in 2010, prior to the disruptive changes that occurred in 2015 and culminated in the “green soup” event, which resulted from a massive *Synechococcus* bloom [30]. Confirming the results above with our time series, the region that shared the highest fraction of vOTUs with this pyrosequenced Mar Menor metagenome was the near-MM region (0.4%). In addition, no vOTUs were shared between the communities of the Mar Menor or the open Mediterranean Sea and the Albufera of Valencia, suggesting that the environmental conditions of the latter, likely due to its lower salinity (an important factor shaping host communities [62]), result in even more distinct viral communities.

### Mar Menor viral communities are mainly driven by strong local events

**Figure 2:**
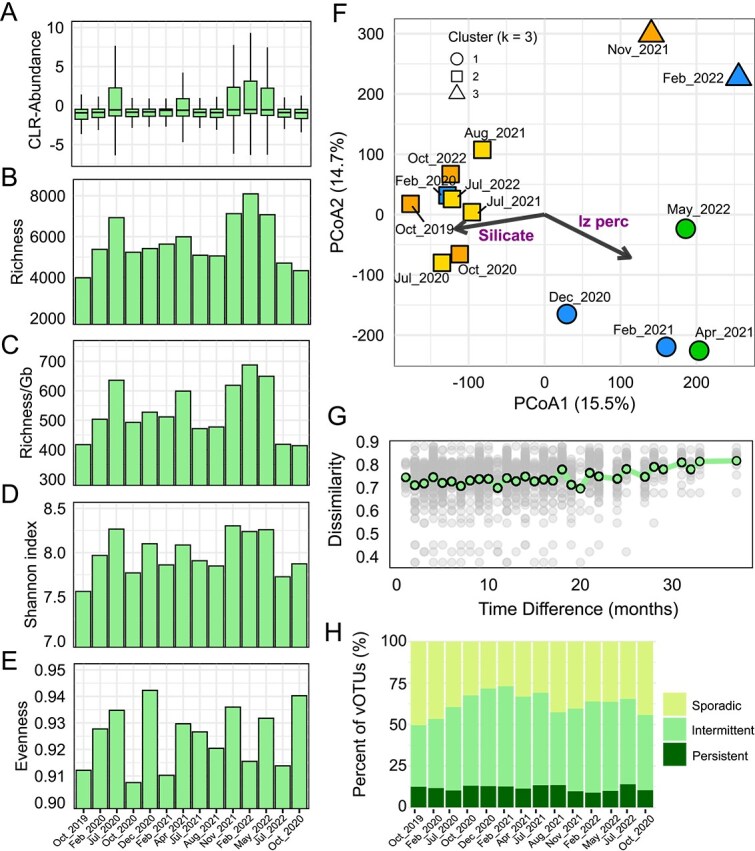
Temporal dynamics of viral communities in the Mar Menor. Boxplot of the CLR-transformed relative abundances of vOTUs (A), and barplots of the observed richness (B), richness normalized per gigabase of metagenomic reads (C), Shannon diversity (D), and Pielou’s evenness (E). (F) Community structure over time shown in the PCoA using a fixed number of three clusters obtained from *k*-means clustering analysis (communities collected in different seasons are represented with different colors: yellow, summer; orange, autumn; blue, winter; and green, spring). Percentages on the axes indicate the proportion of total variance explained by each PCoA dimension. The results of the environmental fitting analysis are indicated by black arrows labeled with purple text (only variables significantly correlated to the community structure are shown). (G) Temporal decay of similarity between communities based on the turnover component of Bray–Curtis dissimilarity values (gray points) and their mean values (light green circles) plotted against pairwise time differences in months. (H) Percentages of dynamic classes of viruses (sporadic, intermittent, or persistent) over time.

Over time, the per-community median CLR-transformed vOTU relative abundances ranged from −0.93 (October 2019) to −0.51 (February 2022) (Fig. 2A; Supplementary Dataset 2). The Friedman test indicated that the vOTU relative abundances differed over time (χ^2^ = 5543.6, df = 13, *P* < 2.2 × 10^−16^) with small effect size (Kendall’s *W* = 0.019). Post hoc analysis demonstrated that all consecutive time points differed (Wilcoxon signed-rank test, *P* < .05), except for a sustained drop between October 2020 and December 2020 (*P* = .121), suggesting strong temporal structuring of viral communities. Notably, vOTU relative abundances were higher in July 2020, November 2021, February 2022, and May 2022 (larger box sizes and higher medians in Fig. 2A). Alpha-diversity metrics followed similar patterns (Fig. 2B–E), with the maximum richness (>6000 vOTUs) and Shannon diversity (>8.2) coinciding with these relative abundance peaks. Such peaks occurred after two pronounced environmental disturbances (Supplementary Fig. 2): (i) the September 2019 torrential rainfall triggering the proliferation of *Synechococcus* and diatoms, initiated in October 2019 and declining in August 2020, as shown by previous 16S rRNA gene amplicon sequencing, flow cytometry, and microscopy data [26, 27] and was accompanied by a first deoxygenation event, and (ii) a second deoxygenation event in August 2021 likely driven by a prolonged period of increased temperatures, during which diatoms became abundant [27, 62, 63]. Both disturbances were associated with reduced water transparency and oxygen levels, along with peaks in chlorophyll and heterotrophic prokaryote abundance. We therefore propose that, with the collapse of dominant host types after the *Synechococcus* prevalence and August 2021, a short-term expansion of host “niches” became available for viral infection, leading to the observed diversity peaks. Additionally, the lack of significant correlations with environmental parameters (Spearman test; *P* > .05; Supplementary Dataset 2) suggests that vOTU relative abundance and alpha-diversity patterns were not directly driven by linear responses to those, but rather by indirect effects mediated via host community restructuring.

The PCoA restricted to the Mar Menor time-series samples (Fig. 2F) explained 30.2% of the variation and revealed certain separation among three sample groups, which were assessed by *k*-means cluster analysis (*k*-means *k* = 3; Supplementary Fig. 4B) and PERMANOVA test (*r*^2^ = 0.28, *P* = .001; Supplementary Dataset 2). Post hoc analyses revealed nonsignificant differences only between clusters 1 vs. 3. While cluster 1 grouped summer and autumn communities (including those collected during the deoxygenation events of October 2019 and August 2021), cluster 3 comprised two consecutive communities from the post-August 2021 period, suggesting that the restructuring of viral assemblages after the latter deoxygenation event was more substantial than that observed after the *Synechococcus* decline (cluster 2). To identify potential environmental drivers structuring the communities, we examined correlations between environmental variables and the ordination space, finding that only two variables correlated significantly with the viral communities: I_z_ percent (a proxy of water transparency; *r*^2^ = 0.53, *P* = .017) and silicate concentration (*r*^2^ = 0.42, *P* = .049) (Fig. 2F). Communities from both October 2019 and August 2021 clustered with those of summer and autumn. This cluster was correlated with lower water transparency, as expected according to their higher picoplankton cell densities (Supplementary Fig. 2). The fact of studying viruses actively interacting with the host (through analysis of metagenomes from cellular size fractions instead of viral fractions) could help explain this strong effect of local disturbances on viral communities [64, 65]. They were also correlated with higher silicate concentrations, which could be explained by increased evaporation and decreased freshwater inputs during summer [66]. These correlations likely reflect indirect trophic and seasonal dynamics that restructure host communities, and consequently, viral assemblages.

Beyond these environmental correlations, we assessed the time variation patterns from the temporal distance-decay of Bray–Curtis dissimilarity, focusing on its turnover (balanced variation) component. The results revealed a significant positive correlation with time (Mantel test, ρ = 0.52, *P* = .000) (Fig. 2G; Supplementary Fig. 6; Supplementary Dataset 2), indicating limited seasonal influence and suggesting that episodic events may act as the main structuring factors. Despite our relatively low number of time points sampled, this result contrasts with the marked seasonality consistently found in open-seawaters viral communities [56, 67–69].

Finally, we examined viral persistence across the 3-year time series by classifying vOTUs into dynamic classes (i.e. virus groups based on their temporal occurrence frequency), following cut-offs similar to those used in previous studies [70]: sporadic (detected in <25% of the time points, i.e. 1–3 out of 14), intermittent (25%–75%, 4–10), and persistent (>75%, 11–14). Overall, the majority of vOTUs were sporadic and intermittent (frequencies of 27.0%–50.5% and 37.1%–60.4%, respectively), and only a small core (8.9%–14.0%) were persistent (Supplementary Dataset 2). This dominance of sporadic viruses in prokaryotic cell fractions has been previously observed in metagenomes of paired viral and cellular size fractions in the San Pedro Ocean time series [64], where persistent viruses were more prevalent in the viral size fractions. Pronounced differences between communities recovered from metagenomes of paired viral and cellular size fractions were also reported in tomato soils, where the rare virosphere was better captured in viromes, while total metagenomes were dominated by the most abundant viruses [65]. Therefore, although the frequencies of dynamic classes observed in our study could reflect response to an unstable environment, they may have differed if focusing on viral fractions. Interestingly, two peaks in relative abundance of sporadic vOTUs coincided with the deoxygenation events of October 2019 and August 2021 (Fig. 2H), suggesting that strong local disturbances select for rare host types. Such a result was supported by pronounced drops, at those dates in the Mar Menor time series, in the number of shared vOTUs with open Mediterranean Sea communities (Supplementary Fig. 5B).

### Lagoon conditions favor copiotroph-infecting phages, with local events shaping virus-host dynamics

**Figure 3:**
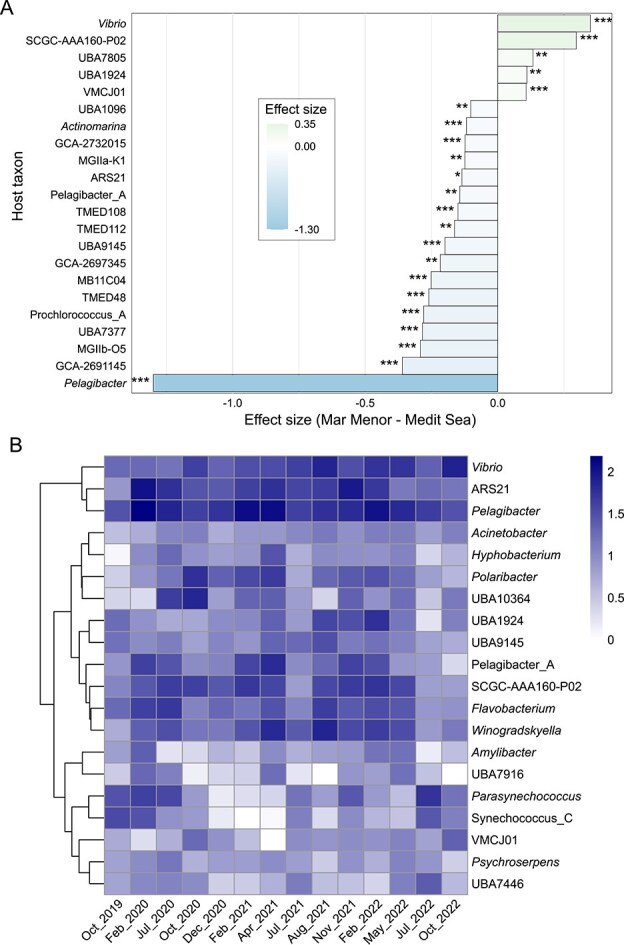
Predicted hosts of viruses. (A) Effect size bar plot showing the difference in the percentage of vOTUs associated with predicted hosts between ecosystems (Mar Menor–Mediterranean Sea); *, **, and *** indicate *P* < .05, *P* < .01, and *P* < .001, respectively. (B) Heatmap of the summed, log-transformed relative abundances of vOTUs predicted to infect the most frequent host taxa across the Mar Menor time series, hierarchically clustered by relative abundance patterns. Host taxa correspond to prokaryotic genera classified according to the Genome Taxonomy Database and predicted using the software iPHoP [44].

We predicted *in silico* the putative hosts for 11.3% (*n* = 2533) of the total vOTUs in the Mar Menor and 16.1% (*n* = 1351) in open waters of the Mediterranean Sea. The contribution of host-associated vOTUs of the sum of relative abundances per viral community ranged from 16% to 53% in the Mediterranean Sea and from 11% to 15% in the Mar Menor. Statistically supported differences (Wilcoxon rank-sum test, *P* ≤ .05, absolute effect size ≥0.1%; Fig. 3A, Supplementary Fig. 7, Supplementary Dataset 3) indicated that the lagoon was enriched in viruses infecting copiotrophic hosts, such as *Vibrio* [71], and taxa associated with marine algae, including the Flavobacteriaceae *Aurantivirga* sp. SCGC-AAA160-P02 [72] and the Phycisphaerae UBA1924. In contrast, open Mediterranean Sea communities were enriched in viruses targeting oligotrophs or streamlined lineages, such as *Pelagibacter*, Prochlorococcus_A, the SAR11-clade-II Pelagibacter_A, *Actinomarina*, and the archaeal genus ARS21 and MGIIa-K1 [73–75]. While oligotrophs (K-strategists) thrive in stable and low-resource aquatic systems [76, 77], copiotrophs (r-strategists) are especially suited to habitats with high nutrient flux [78, 79]. Therefore, we presume that both the predominance of copiotrophic hosts and their high metabolic activity in the Mar Menor contributed to the observed higher vOTU relative abundances. This pattern is expected to partly reflect bias arising from the use of cellular rather than viral size fractions, which captures abundant intracellular viruses but not the entire viral community [64, 65]. Substantially large effect size was observed in *Pelagibacter* (SAR11 clade I) phages over those infecting other host genera, including Pelagibacter_A (SAR11 clade II), which may reflect an additive effect of undescribed members within *Pelagibacter*, as this genus contains high microdiversity [80, 81]. Although Pelagibacteraceae genera are known for their dominance in oligotrophic waters, it is possible that the lower effect size observed for Pelagibacter_A phages may point to better adaptation to eutrophic waters, as diverse ecological strategies have been reported within the SAR11 clade II [82].

The analysis of host-specific vOTU relative abundances in the Mar Menor over time (Fig. 3B) revealed three emerging pattern clusters: (i) consistently abundant viruses infecting *Pelagibacter*, ARS21, and *Vibrio*; (ii) lower-abundance viruses mostly associated with Flavobacteriaceae (*Polaribacter, Winogradskyella, Flavobacterium*, and *Aurantivirga* sp. SCGC-AAA160-P02), possibly derived from opportunistic host shifts in response to changes in availability of specific resources [83]; and (iii) viruses targeting *Synechococcus* and other co-occurring hosts, such as *Amylibacter* [84] and Flavobacteriaceae (*Psychroserpens* and UBA7446). The host genera predicted from Mar Menor viral contigs broadly resembled (*sensu lato*) bacterioplankton taxa previously identified by rRNA gene sequencing [27]. That study reported dominance of the SAR11 clade (here, explaining pelagiphages) and non-Pseudomonadales Gammaproteobacteria (vibriophages), with *Synechococcus* peaking during the same periods when their cyanophages were abundant (October 2019–July 2020, July–November 2021, and July–October 2022). Notably, viral patterns of *Synechococcus*_C and *Parasynechococcus* correlated with *Synechococcus* cell density (Spearman test, ρ ≈ 0.9; Supplementary Dataset 3), indicating a rapid phage communities response to host shifts [85]. Given their potential as biocontrol agents [86], these findings provide preliminary support for using cyanophages to manage *Synechococcus* blooms, pending further optimization for efficacy and scale.

### Environmental conditions of the lagoon do not clearly influence the level of temperate viruses

**Figure 4:**
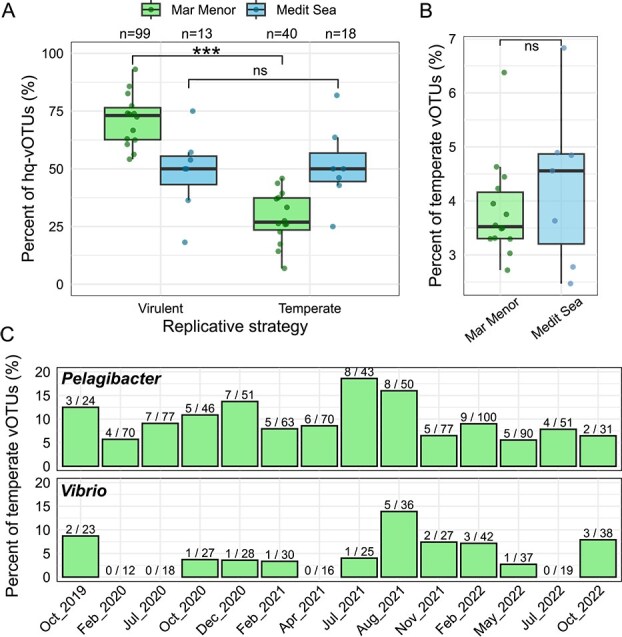
Frequency of viruses associated with replicative strategies. (A) Percentage of vOTUs with high-quality genomes (hq-vOTUs) classified as virulent and temperate (predicted using BACPHLIP [45]) per sample in each environment, with the absolute numbers of total vOTUs displayed at the top. (B) Percentage of temperate vOTUs per sample in each environment based on the presence of virus-like recombinase genes. (C) Frequency of temperate vOTUs (virus-like recombinase genes) predicted to infect *Pelagibacter* and *Vibrio* in the Mar Menor. The fractions indicated on each bar represent the number of temperate vOTUs over the total detected per time point.

There is an ongoing debate about the influence of the host metabolic status on lysogeny [21–24, 87], with two models being proposed: (i) the low-density refugium model, which suggests that lysogeny is favored when host density is low, and (ii) the piggyback-the-winner model, in which lysogeny is favored under high microbial densities. To assess whether the eutrophic conditions of the Mar Menor influence the reproductive strategy (virulent vs. temperate), we annotated high-quality genomes of vOTUs from this lagoon and the open Mediterranean Sea. The significantly higher frequency of virulent over temperate vOTUs in the Mar Menor (Wilcoxon rank-sum test, *P* = .000) (Fig. 4A) does not, in principle, support the piggyback-the-winner model. Nevertheless, the lack of a significant difference between virulent and temperate vOTUs in the oligotrophic waters of the open Mediterranean Sea (*P* = 1) does not support either lysogeny as a refugium strategy. To expand our analysis beyond the few high-quality genomes, we screened for recombinase genes (hallmarks of temperate viruses) in all viral contigs. No significant differences (*P* > .05) in the temperate vOTU frequency between the Mar Menor and the open Mediterranean Sea, both per whole-community (Fig. 4B) and per-host levels (to avoid “dilution” effect) (Supplementary Dataset 4), suggest that the lagoon eutrophic conditions neither promote nor diminish lysogeny under the environmental regimes sampled here and within the limits of our approach to detect temperate viruses. Importantly, the analytical strategy followed relies exclusively on marker gene detection in metagenomes and lacks complementary metatranscriptomic data to assess gene expression. Moreover, accumulating evidence indicates that prokaryotic viruses adopt alternative replicative strategies beyond the classical lysis–lysogeny dichotomy (such as pseudolysogeny or chronic infection) [88] that are not readily captured by marker gene-based approaches.

We explored the influence of the lagoon environmental variability on lysogeny by studying the frequency of temperate vOTUs associated to the dominant phage groups, pelagiphages and vibriophages, over time (Fig. 4C). Although no significant correlations were found either among them or with environmental factors (Supplementary Dataset 4), temperate pelagi- and vibriophage frequencies showed considerable variation (5.6%–18.6% and 2.7%–13.9%, respectively) and peaked with strong local events (October 2019 and August 2021), though not exclusively. As previously suggested [12, 24], episodic disturbances causing fluctuations in host physiology could hinder viral replication and thus select for temperate viruses, potentially conferring, in turn, adaptive advantages to the host.

### The distribution of auxiliary viral genes was strongly shaped by ecosystem type and local events

**Figure 5:**
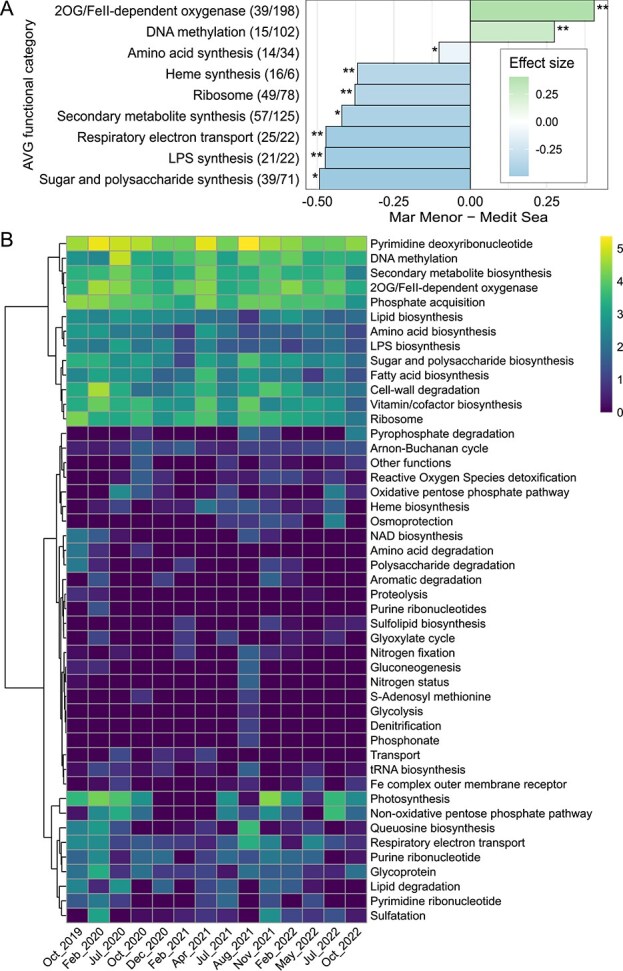
Metabolic reprogramming by viruses. (A) Effect size bar plot showing the difference in the percent of vOTUs with AVG functions between ecosystems (Mar Menor–Mediterranean Sea); ^*, **^, and ^***^ indicate *P* < .05, *P* < .01, and *P* < .001, respectively. (B) Heatmap showing relative abundance of vOTUs carrying AVG of each functional category in the Mar Menor over time.

In addition to viral core functions, viruses can influence metabolism, physiology, and regulation of host cells through AVGs, a term recently proposed [89]. To assess this type of viral footprint across the Mar Menor and the open Mediterranean Sea, we functionally annotated viral genomes (Supplementary Data 5), identifying 2357 AVGs in 1754 vOTUs (out of a total of 30 637 vOTUs; 5.7%) linked to 51 functional categories. When comparing the frequency of vOTUs carrying these genes across ecosystems, DNA methyltransferases (Wilcoxon test rank-sum, *P* = .001) and 2-oxoglutarate (2OG)/Fe(II)-dependent oxygenases (*P* = .002) were significantly more prevalent in the lagoon (Fig. 5A, Supplementary Fig. 8). Methylation of viral DNA have been previously associated with viral evasion of host antiviral defenses [90, 91]. Meanwhile, discerning the functional relevance of viral 2OG/Fe(II)-dependent oxygenases is less clear, given their involvement in disparate physiological roles in bacteria [92]. AVGs encoding this domain have been proposed to be involved in DNA repair and regulation of nitrogen metabolism [93]. To gain deeper insight into their specific function, we built a sequence similarity network including our viral proteins and reference proteins annotated as 2OG/Fe(II)-dependent oxygenases (Supplementary Fig. 9). Most viral proteins (92.9% in the Mar Menor and 83.7% in the open Mediterranean Sea; Supplementary Data 5) exclusively matched a *Shewenella baltica* protein carrying the PiuC domain, which has been proposed to participate in iron uptake [94, 95]. This signature was present in 81.8% and 76.7% of these oxygenases in the lagoon and the open sea, respectively. In contrast, AVG functionally annotated as being involved in processes such as (i) energy obtention via respiratory chain factors (heme biosynthesis, respiratory chain), (ii) increased protein biosynthesis (amino acid biosynthesis and ribosome biogenesis), (iii) secondary metabolite production, and (iv) modification of host-cell surface (lipopolysaccharide and polysaccharide biosynthesis) to prevent attachment of other viruses [96] (*P*-values < .05) were more prevalent in the open sea.

We also studied the relative abundance patterns of function-specific AVGs over time (Fig. 5B). First, a strong negative correlation was found between 2OG/Fe(II)-dependent oxygenases and chlorophyll *a* concentration (Spearman test, ρ = −0.9, *P* = .000) (Supplementary Data 5). Marine photosynthesis is strongly dependent on iron [97] and *piuC*-carrying genes are linked to picocyanobacterial responses to iron stress [98]. Nevertheless, this correlation does not allow inferring a mechanistic link, particularly given that the bioavailability of iron remains to be determined despite evidence of metal pollution in the lagoon [99]. Second, AVGs involved in heme (iron-protoporphyrin IX) biosynthesis, which is essential for respiratory chain and redox reactions, were negatively correlated with oxygen levels (ρ = −0.9, *P* = .015), which might reflect host energy production during episodic deoxygenation.

Beyond correlations, three clusters of AVG relative abundance patterns were identified (Fig. 5B): (i) nearly constant genes, likely encoding particularly relevant viral functions (e.g. deoxyribonucleotide and protein biosynthesis, or DNA methylation) (upper section of the heatmap); (ii) patterns resembling those of *Synechococcus* cell density (Supplementary Fig. 2) and *Synechococcus*-associated vOTU relative abundance (Fig. 3B), and comprising AVGs linked to photosynthesis and nonoxidative pentose phosphate pathway (lower section of the heatmap); and (iii) patterns with lower-abundance and occurrence (central section of the heatmap). Interestingly, the peak of AVGs related to NAD+ biosynthesis, amino-acid and polysaccharide degradation in October 2019 might be a response to the drastic water changes after the torrential rainfalls. The deoxygenation event of August 2021 was accompanied by AVGs targeting (i) nitrogen pathways typically performed under low oxygen (nitrogen fixation and denitrification) and (ii) NADPH and NADH oxidation pathways (deoxyribonucleotide and queuosine biosynthesis, and gluconeogenesis). Altogether, our results suggest that the distribution of AVG may be influenced by the environmental conditions [100]. However, it should be noted that the AVG patterns observed here are also expected to be affected by the use of metagenomes of cellular size fractions [64, 65]. Moreover, the potential ecological roles of these AVGs should be interpreted with caution, as they are solely based on metagenome annotation.

## Conclusions

This study provides a metagenomic assessment addressing multiple aspects of prokaryotic DNA virus ecology in a highly impacted coastal lagoon, using both comparative and temporal frameworks. Compared to those in the open Mediterranean Sea, the lagoon viral communities showed not only higher vOTU relative abundances and diversities, but also compositional divergence, with preferential infection of copiotrophic hosts and carrying more AVGs encoding DNA methyltransferases and 2OG/Fe(II)-dependent oxygenases. Such trends may stem not only from eutrophication and strong physicochemical dynamism, but also from the limited water exchange with the open sea. Temporally, viral communities showed no clear seasonality but were correlated with water transparency and silicate concentration. Overall, the viral assemblage appeared strongly impacted by local events, a signal reflected in multiple viral features. We found no conclusive evidence, either comparatively or temporally, regarding viral replicative strategies, underscoring the complexity of the factors regulating the lysis–lysogeny switch beyond host cell density and metabolic activity.

This study emphasizes the importance of investigating viruses to better predict ecosystem responses to eutrophication, an increasingly common stressor. We further underscore the value of incorporating time-series analyses in coastal lagoons, given their substantial temporal variability. Future work should include extracellular virion sampling to better capture rare taxa and noninfecting viruses, thereby expanding the recovered viral genome space. In addition, applying long-read sequencing technologies would greatly aid in identifying virus–host junctions of prophages, and consequently, in understanding the factors that drive lysogeny.

## Supplementary Material

ycag110_Supplementary_materials

## Data Availability

The raw sequencing reads obtained in this study were deposited in the European Nucleotide Archive (http://www.ebi.ac.uk/ena) under project accession PRJEB101825. Raw and processed tables are available in Supplementary Data 1–5 (description available in Supplementary information). R scripts are available in GitHub at https://github.com/virosphaera/Mar_Menor_Ecology_prokaryotic_viruses. Contig sequences of the prokaryotic viruses analyzed across all marine environments and input datasets to reproduce statistics and figures in this study have been deposited in Zenodo (version 3; 10.5281/zenodo.18700989).
