# Supplementary material for: Ecology of prokaryotic DNA viruses in a highly impacted coastal lagoon revealed through comparative and temporal metagenomics
Source: ISME Commun. 2026 Apr 21;6(1):ycag110. doi: 10.1093/ismeco/ycag110 (PMC13184970; doi:10.1093/ismeco/ycag110)
Supplement: ycag110_Supplementary_materials [file ycag110_supplementary_materials.zip › Supplementary_information_after_provisional_acceptance.docx]

**Supplementary Information for Domínguez-Huerta *et al*. Ecology of prokaryotic DNA viruses in a highly-impacted coastal lagoon revealed through comparative and temporal metagenomics**

Supplementary Figures 1-9 captions

**Supplementary Figure 1**. Map showing the sampling stations for all metagenomes analysed in this study.

**Supplementary Figure 2**. Temporal scale of sampling in the Mar Menor. **Supplementary Figure 3**. Summarized schematics of the experimental procedures and bioinformatic workflow for viral identification.

**Supplementary Figure 4**. Variation of the average silhouette width (Y-axis) with respect to the number of clusters (X-axis) derived from k-means clustering of viral community composition data.

**Supplementary Figure 5**. Log-transformation of the shared vOTU percentage (according to the Sørensen–Dice coefficient; see Methods) between communities.

**Supplementary Figure 6**. Total Bray-Curtis dissimilarity (top) and its abundance-driven component (bottom), plotted as a function of temporal distance (in months) between viral communities.

**Supplementary Figure 7**. Boxplots of vOTU percentages per host genus across samples from the Mar Menor and Mediterranean Sea.

**Supplementary Figure 8**. Boxplots of vOTU percentages per AVG functional category across samples from the Mar Menor and Mediterranean Sea.

**Supplementary Figure 9**. Protein sequence similarity network (SSN) of 2-oxoglutarate/Fe(II)-dependent oxygenases derived from AVGs of this study and reviewed UniProtKB reference proteins.

Supplementary Datasets captions

**Supplementary Dataset 1**. Dataset with information on samples, environmental measurements, metagenome statistics, viral contigs, viral taxonomy inferred by vConTACT3, vOTU clustering and their normalized relative abundances, alpha-diversity metrics, and statistical analyses related to the comparative analyses.

**Supplementary Dataset 2**. Dataset with information the temporal analyses on community structure, envfit analysis, Bray-Curtis distance partitioning and their statistical trends over time, dynamic classes, and shared vOTUs between communities.

**Supplementary Dataset 3**. Dataset with information on the putative hosts predicted for viral contigs and vOTUs by iPHoP, effect size analyses and Wilcoxon test for the Med-MM comparison, and Spearman correlation test for the MM over time.

**Supplementary Dataset 4**. Dataset with information on the prediction of the viral replicative strategies based on BACPHLIP and screening of viral recombinases, vOTU percentages per replicative strategy across samples from the Mar Menor and Mediterranean Sea, vOTU percentages per replicative strategy and host genus across samples from the Mar Menor and Mediterranean Sea, respective Wilcoxon statistical tests, vOTU percentages per replicative strategy in *Pelagibacter* and *Vibrio* in the Mar Menor over time, and Spearman correlation tests.

**Supplementary Dataset 5**. Dataset with information on the functional annotation of the viral contigs, the identified AVGs and their functional categories, vOTU percentage per AVG functional category and sample across the Mar Menor and the Mediterranean Sea, the respective Wilcoxson statistical test, and Spearman correlation test of the vOTU relative abundances aggregated by the AVG functional category in the Mar Menor over time and the environmental factors.

**Supplementary Figures 1−9**


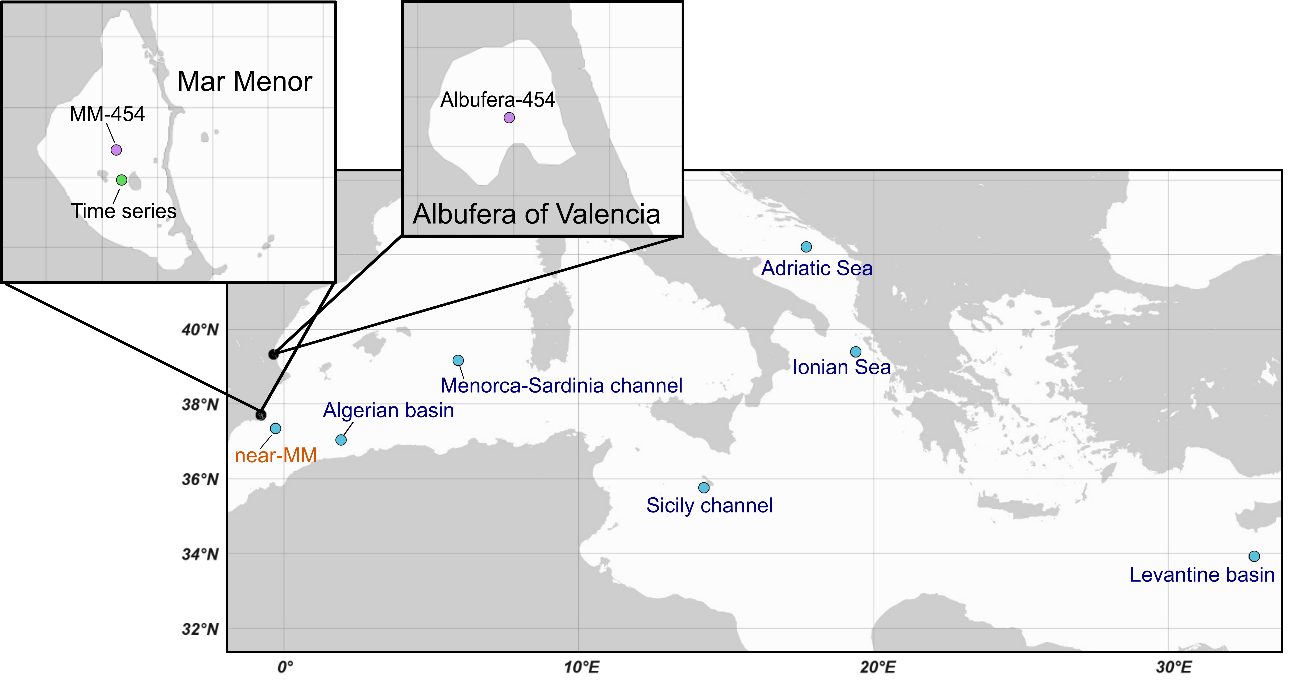


**Supplementary Figure 1.** Map showing the sampling stations for all metagenomes analysed in this study. The insets show the coastal lagoons (Mar Menor and the Albufera of Valencia). In the insets, the green point indicates the sampling station in the Mar Menor corresponding to the time series reported in our study (“Time series”) while the purple points represent the pyrosequenced metagenomes sampled in the Mar Menor and the Albufera of Valencia in 2010 and reported by Ghai *et al*. (2012) (“MM-454” and “Albufera-454”, respectively). The sampling stations of previously published datasets from open waters of the Mediterranean Sea (blue points) are shown in the larger map, where the orange text indicates the sampling station of the metagenome reported by López-Pérez *et al*. (2017) (the open-sea region closest to the Mar Menor, “near-MM”) and the blue text indicate the six metagenomes from *Tara* Oceans campaign across the Mediterranean Sea.


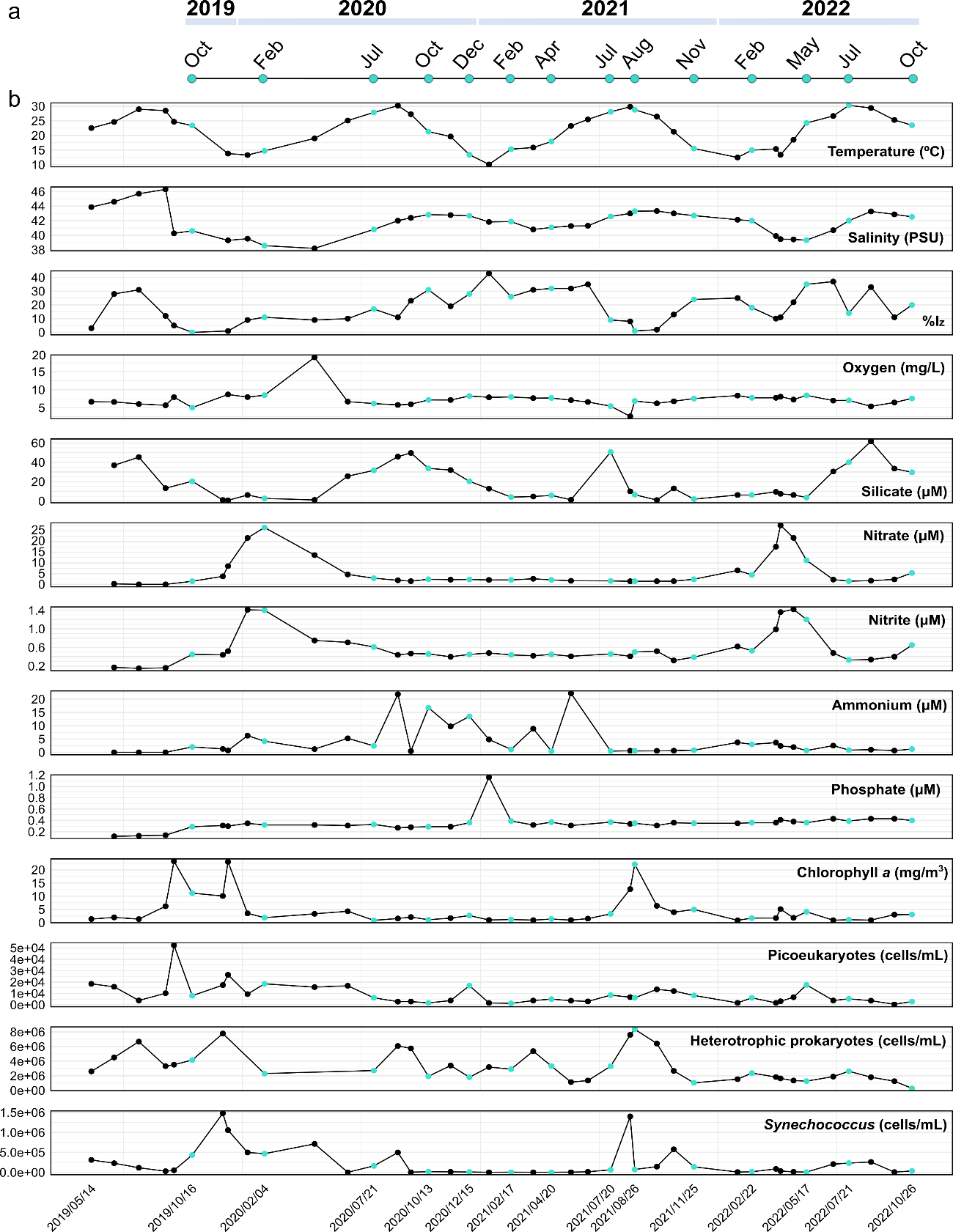


**Supplementary Figure 2**. Temporal scale of sampling in the Mar Menor. (**a**) Time series showing the dates when picoplankton metagenomes were collected (in turquoise). (**b**) Variation of the hydrological variables (temperature, salinity, water transparency, oxygen), concentration of nutrients (nitrate, nitrite, ammonium, phosphate), chlorophyll *a* concentration, and picoplankton cell densities (picoeukaryotes, heterotrophic prokaryotes, and *Synechococcus*) from May 2019 to October 2022. Turquoise points in all panels indicate time points when metagenomic data were generated.


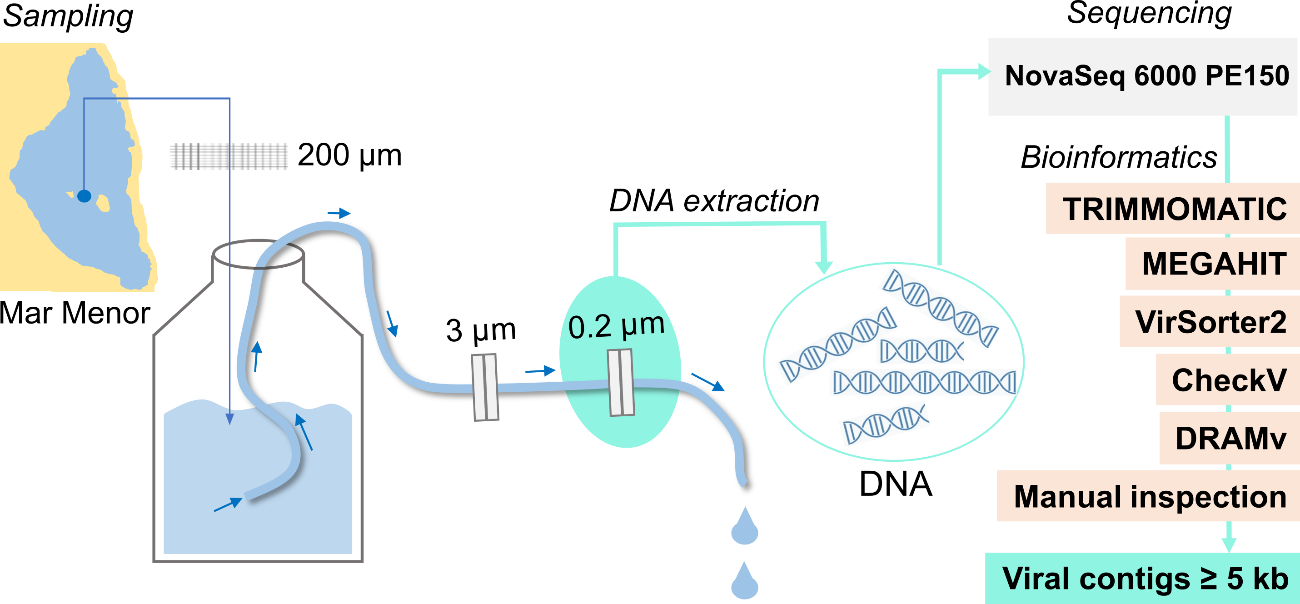


**Supplementary Figure 3**. Summarized schematics of the experimental procedures and bioinformatic workflow for viral identification. Water samples were pre-screened by a mesh of 200 μm and sequentially filtered through 3 μm and 0.2 μm pore-size polycarbonate membrane filters using a peristaltic pump. The DNA was extracted from the filtered bacterioplankton using the DNeasy PowerSoil Pro Kit and sequenced for shotgun metagenomic on an Illumina NovaSeq 6000 PE150 platform. After assembling quality-checked sequencing reads, only contigs of at least 5 kb were annotated with several bioinformatic software for viral identification.


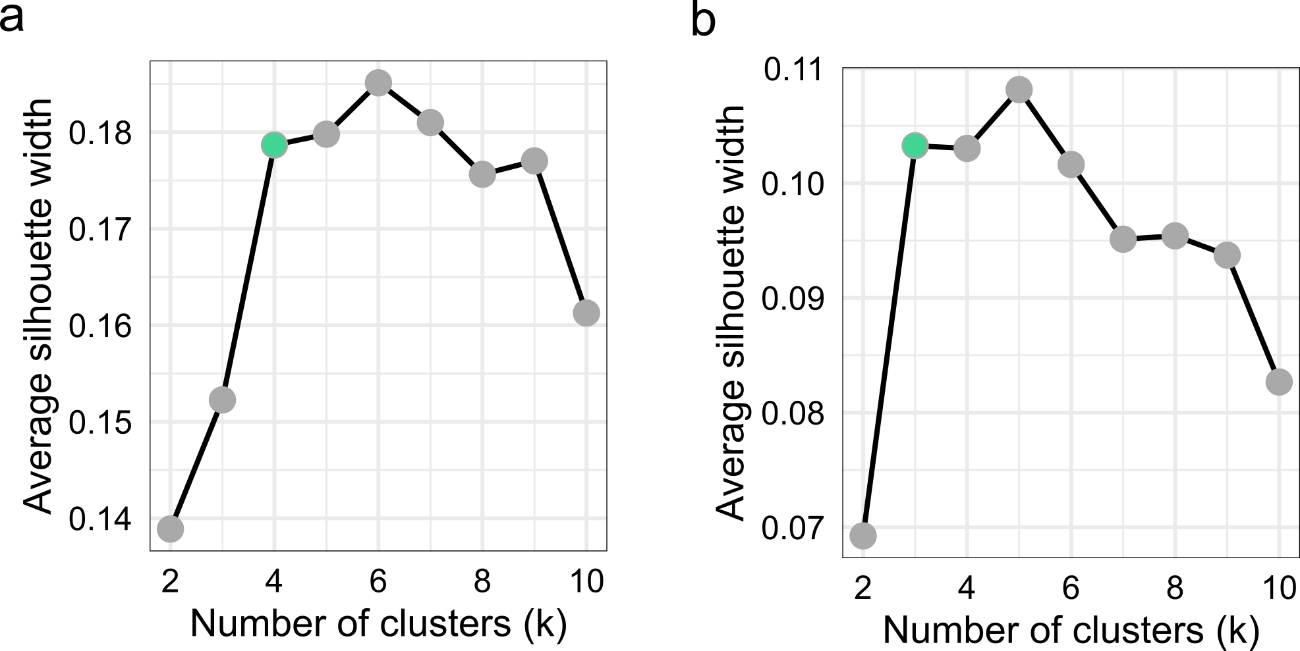


**Supplementary Figure 4**. Variation of the average silhouette width (Y-axis) with respect to the number of clusters (X-axis) derived from k-means clustering of viral community composition data. The silhouette width indicates the strength and coherence of sample grouping, with higher values reflecting more distinct and well-separated clusters. We selected the minimum number of clusters with the maximum average silhouette width (point in turquoise) for the principal coordinate analysis. (**a**) Comparison between viral communities from the Mar Menor and the Mediterranean Sea. (**b**) Temporal variation of viral communities within the Mar Menor.


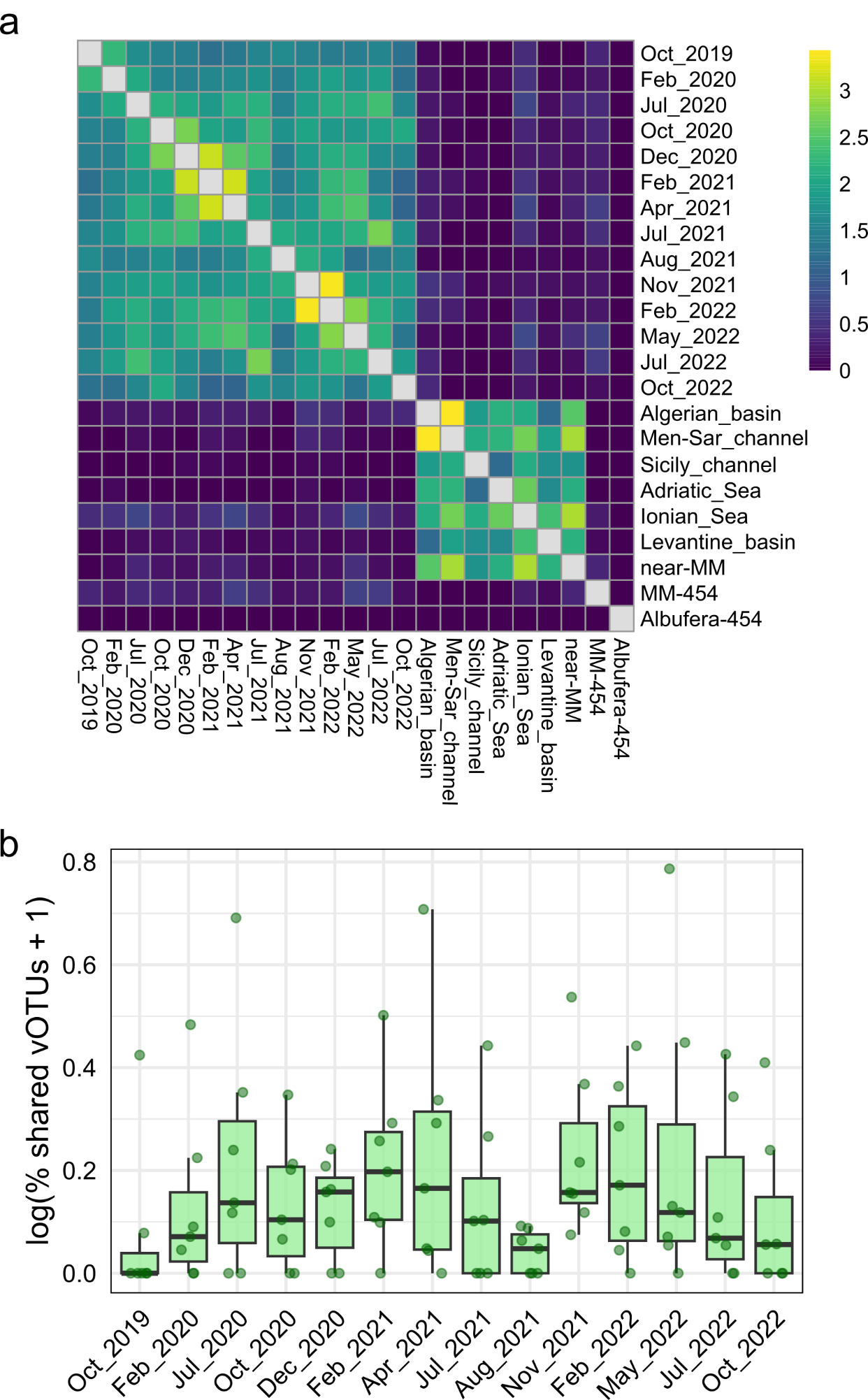


**Supplementary Figure 5.** Log-transformation of the shared vOTU percentage (according to the Sørensen-Dice coefficient; see Methods) between communities. (**a**) Heatmap showing the log-transformed percentage between samples across the Mar Menor (MM) time series (indicated by dates), the Mediterranean Sea (near-MM, Algerian basin, Menor-Sardinia channel, Sicily channel, Adriatic Sea, Ionian Sea, and Levantine basin), and the pyrosequenced metagenomes from Ghai *et al*. (2012) (“MM-454” and “Albufera-454”). (**b**) Boxplot showing the log-transformed percentages between samples from the Mar-Menor time series and regions of the open Mediterranean Sea.


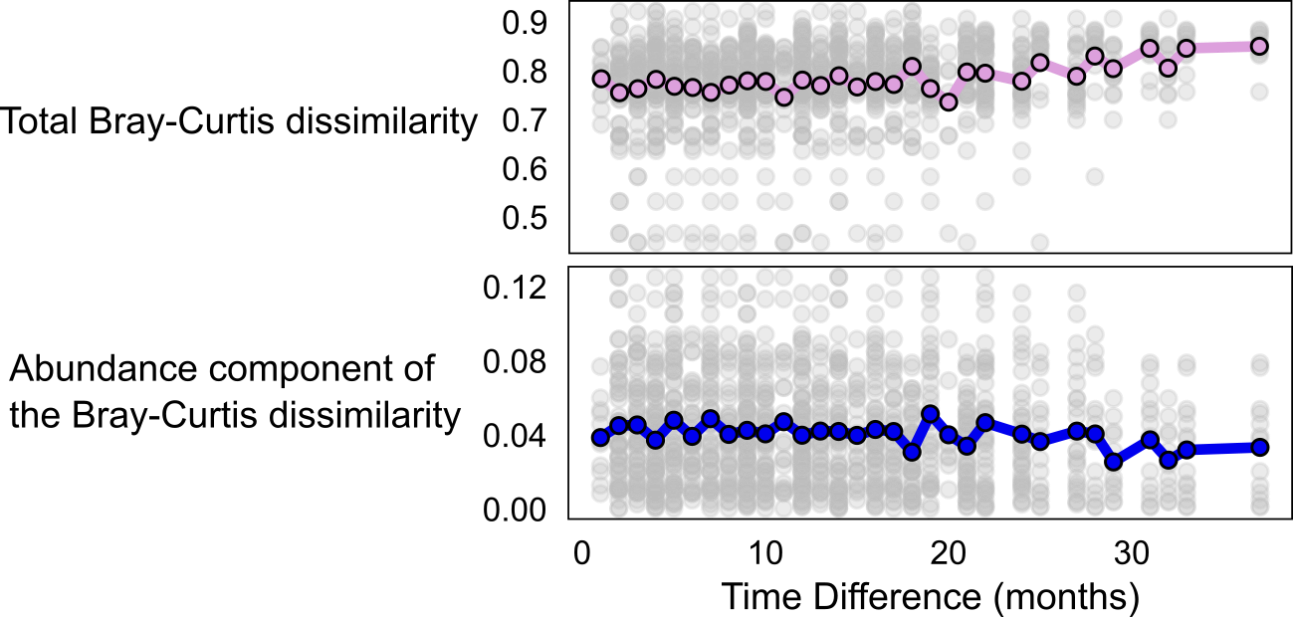


**Supplementary Figure 6**. Total Bray-Curtis dissimilarity (top) and its abundance-driven component (bottom), plotted as a function of temporal distance (in months) between viral communities.


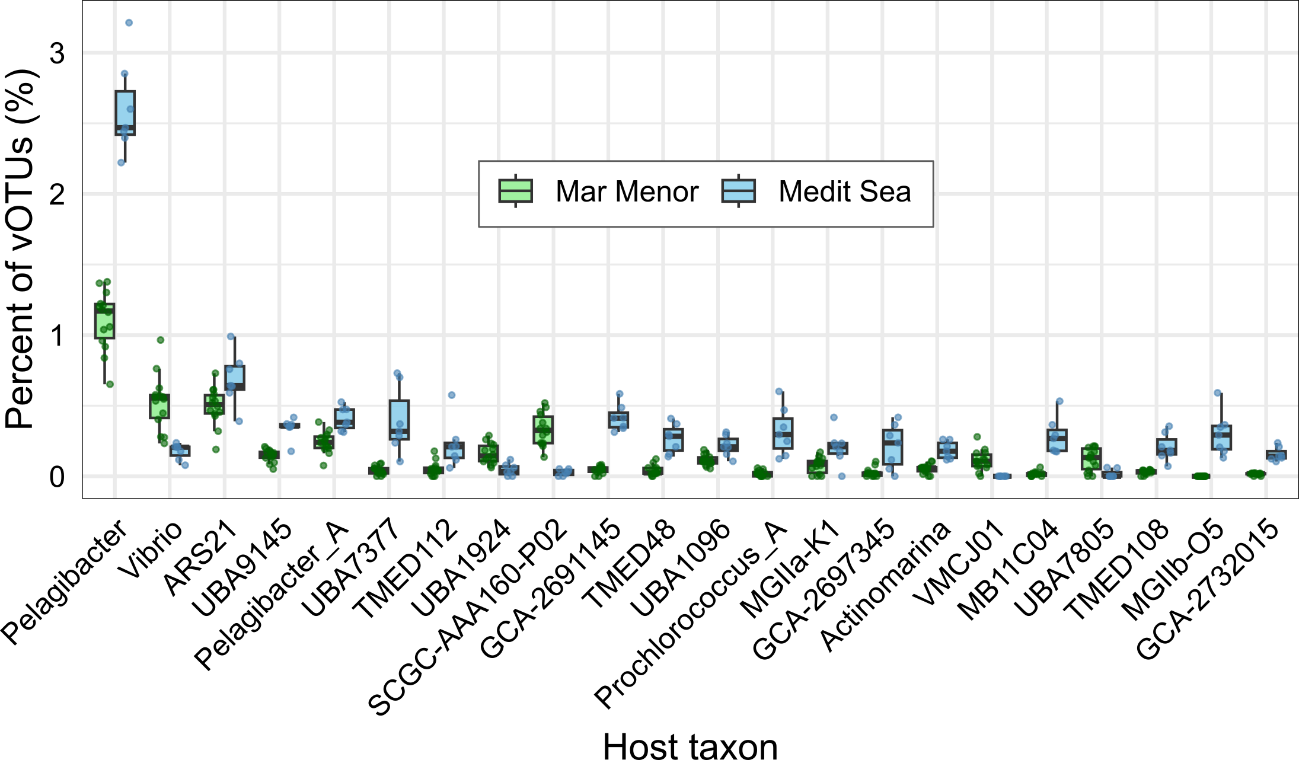


**Supplementary Figure 7**. Boxplots of vOTU percentages per host genus across samples from the Mar Menor and Mediterranean Sea. Only cases for which there was a significant difference (*p≤*0.05) with an absolute effect size of at least 0.1% are shown.


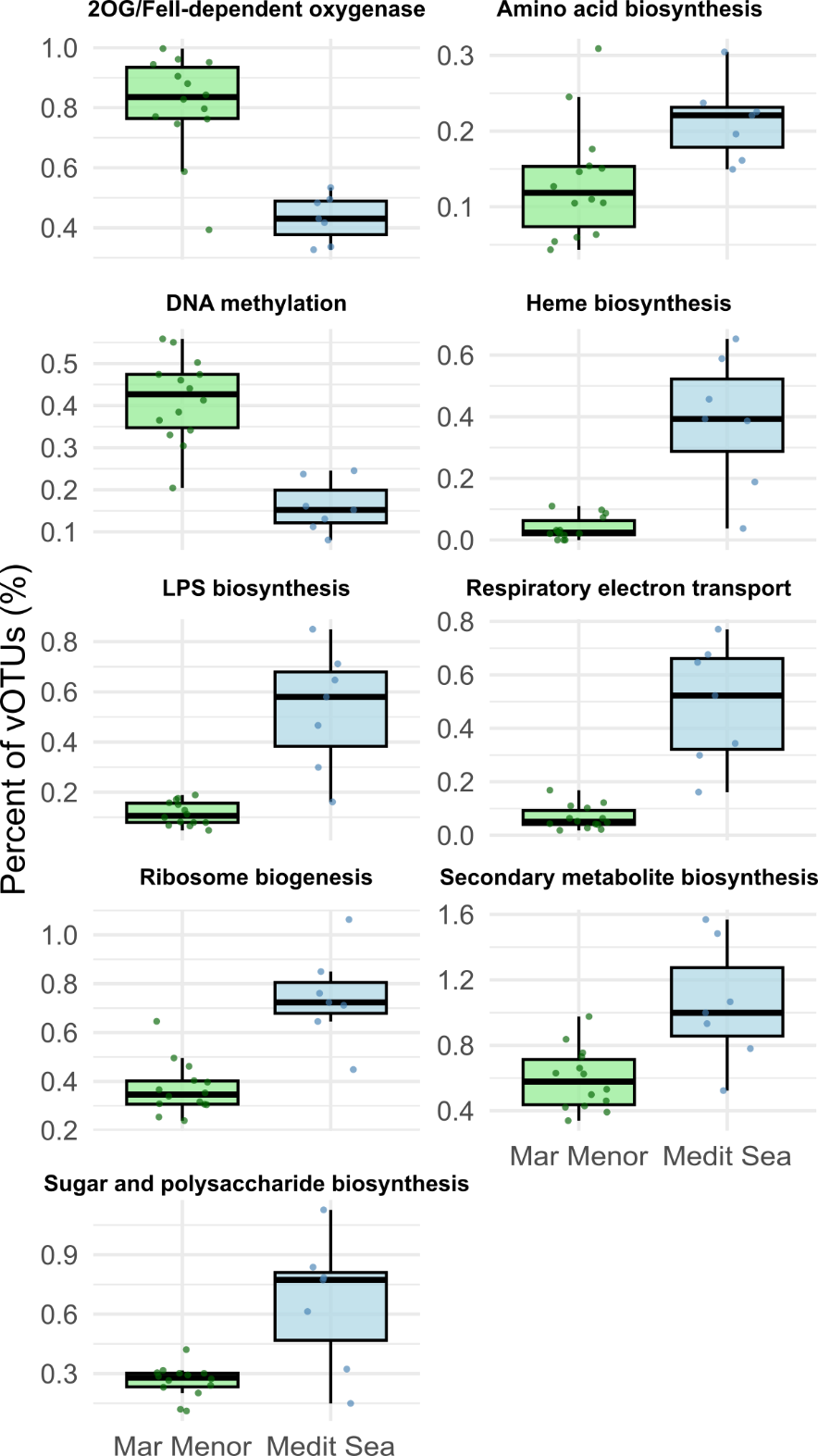


**Supplementary Figure 8**. Boxplots of vOTU percentages per AVG functional category across samples from the Mar Menor and Mediterranean Sea. Only cases for which there was a significant difference (*p ≤* 0.05) with an absolute effect size of at least 0.1% were shown here.


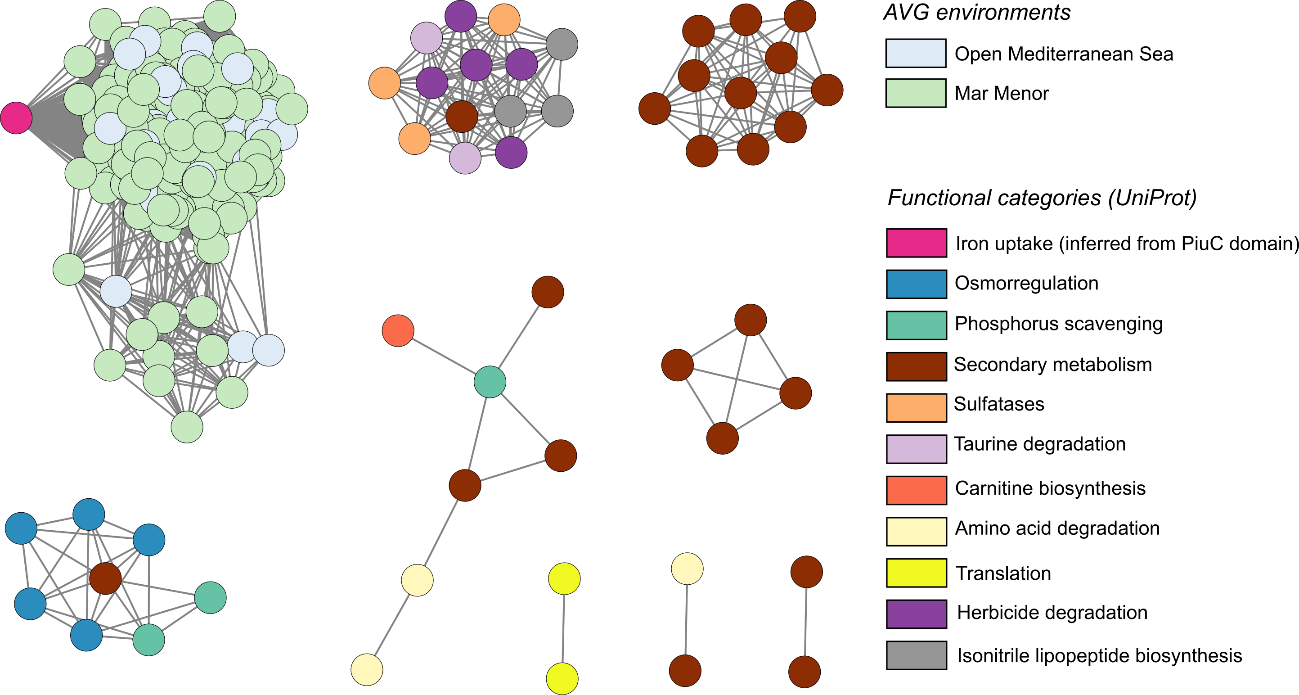


**Supplementary Figure 9**. Protein sequence similarity network (SSN) of 2-oxoglutarate/Fe(II)-dependent oxygenases derived from AVGs of this study and reviewed UniProtKB reference proteins. The SSN was generated using BLASTP and visualized in Cytoscape. Edges connect protein pairs meeting the thresholds e-value ≤ 10^-5^ and bit score ≥ 37 (see Methods). Nodes are colored either by sample environment (Mar Menor vs. regions of the open Mediterranean Sea) or by the UniProtKB functional category (**Supplementary Data 5**). The function of the reference protein Y3634_SHEB5 (iron uptake) was assigned by detecting a PiuC domain via searches against the NCBI Conserved Domain Database (**Supplementary Data 5**).

**Supplementary discussion**

***Methodological considerations***

To study the impact of the multiple particularities of the Mar Menor coastal lagoon (higher temperature, salinity, and nutrient levels, smaller size, partial isolation, frequent local disturbances, etc.) on its viral communities collected from October 2019 to October 2022, we compared those against the ones extracted from previously obtained picoplankton metagenomes in surface waters of the Mediterranean Sea. Although all datasets were processed using the same bioinformatic pipeline, residual methodological differences (e.g., water sampling, DNA extraction, library preparation, sequencing platform and depth; **Supplementary Dataset 1**) may introduce biases that can be mistaken for biological signal, yielding artefactual differences. We briefly describe these methodological differences introducing potential biases as follows. First, the considerably smaller **water volumes** sampled in the Mar Menor (0.6−1 L) would be expected to increase the risk of stochastic dominance of fewer viral taxa, underrepresenting diversity compared to the much larger volumes collected from the Mediterranean Sea (100 L for the *Tara* Oceans expedition [1] and 200 L for the near-MM sample [2, 3]). Second, although the comparison included only picoplankton fractions, different **fraction size ranges** were utilized for the *Tara* Oceans (0.22−1.6 μm) and near-MM (0.22−5 μm) samples compared to our own fraction (0.22−3 μm), which may incorporate higher picoplankton biomass carrying more infecting viruses. Third, while our **DNA extraction** was performed with a commercial silica membrane-based kit optimized for environmental samples (DNeasy PowerSoil Pro Kit), DNA from the open sea (both *Tara* and near-MM) was extracted using a custom protocol based on enzymatic and chemical lysis, phenol-chloroform extraction, followed by ultrafiltration-based concentration [4, 5]. The latter DNA extraction strategy has been reported to outperform the former [6], likely increasing the recovery of more complete and diverse viral communities from the Mediterranean samples analysed here. Fourth, our metagenomic libraries were sequenced on an Illumina NovaSeq 6000, producing 2×150-bp paired-end reads. In contrast, the *Tara* datasets were generated on an Illumina HiSeq 2000 (2×100 bp), and the near-MM sample on an Illumina HiSeq 4000 (2×150 bp). Given that there is better recovery of assembled contigs and more limited chimericity of viral genomes when using longer Illumina reads [7, 8], one could expect that such difference would have an important impact on the viral communities reconstructed from the *Tara* samples. Finally, one key limitation lies in the mismatch of **spatial and temporal scales** between the Mar Menor (temporal fluctuations) and the Mediterranean Sea (single-time points of disparate regions) samples.

Despite these differences at multiple methodological steps, we argue that our results mostly express biological differences between the two ecosystems given the following reasoning. First, we argue that the observed higher vOTU relative abundance and diversity in the communities recovered the Mar Menor with strong significance (**Fig. 1**) spoke against the putative bottlenecks derived from at least three out of the five potential biases described above (water volume, size fractioning, and DNA extraction). Second, the potential detrimental effect derived from shorter reads used in the *Tara* samples may have been overridden by this lower vOTU relative abundance and diversity, as this pattern was also observed in the near-MM sample. In addition, after quality filtering of the paired-end reads, the total number of read nucleotides per sample was of the same order of magnitude across datasets, with overlapping ranges (lagoon: 9.54×10^9^–1.51×10^10^ nts; *Tara*: 7.76×10^9^–3.72×10^10^ nts; near-MM: 2.30×10^10^ nts; **Supplementary Dataset 1**), suggesting broadly comparable sequencing depths. Finally, we argue that, although comparing a temporal series against single-time points of disparate regions certainly precludes direct temporal comparisons, the higher vOTU relative abundance and richness was detected consistently over a time period including significantly varying environmental conditions in the lagoon (accompanied by very different compositions of viral communities), suggesting that the observed differences are not merely due to temporal variability. Rather than asserting direct comparability, our analyses contrast the Mar Menor with the Mediterranean Sea to test how the unique characteristics of the lagoon may have shaped the striking differences observed in its viral communities.

**References cited in the Supplementary information**

1. Pesant S, Not F, Picheral M, Kandels-Lewis S, Le Bescot N, Gorsky G *et al*. Open science resources for the discovery and analysis of Tara Oceans data. *Sci Data* 2015;**2**:150023. https://doi.org/10.1038/sdata.2015.23

2. Haro-Moreno JM, López-Pérez M, de la Torre JR, Picazo A, Camacho A, Rodríguez-Varela F*.* Fine metagenomic profile of the Mediterranean stratified and mixed water columns revealed by assembly and recruitment. *Microbiome* 2018;**6**:128. https://doi.org/10.1186/s40168-018-0513-5

3. López-Pérez M, Haro-Moreno JM, González-Serrano R, Parras-Moltó M, Rodríguez-Varela F. Genome diversity of marine phages recovered from Mediterranean metagenomes: Size matters. *PLOS Genetics* 2017;**13**:e1007018. https://doi.org/10.1371/journal.pgen.1007018

4. Alberti A, Poulain J, Engelen S, Labadie K, Romac S, Ferrera I *et al*. Viral to metazoan marine plankton nucleotide sequences from the Tara Oceans expedition. *Sci Data* 2017;**4**:170093. https://doi.org/10.1038/sdata.2017.93

5. Martin-Cuadrado A-B, Rodríguez-Valera F, Moreira D, Alba JC, Ivars-Martínez E, Henn MR *et al*. Hindsight in the relative abundance, metabolic potential and genome dynamics of uncultivated marine archaea from comparative metagenomic analyses of bathypelagic plankton of different oceanic regions. *ISME J* 2008;**2**:865–886. https://doi.org/10.1038/ismej.2008.40

6. Lima Â, Sousa LGV, Macedo F, Muzny CA, Cerca N. Assessing recovery rates of distinct exogenous controls for gDNA extraction efficiency using phenol-chloroform or silica-column based extractions. *J Microbiol Methods* 2022;**203**:106607. https://doi.org/10.1016/j.mimet.2022.106607

7. Mende DR, Waller AS, Sunagawa S, Järvelin AI, Chan MM, Arumugam M *et al*. Assessment of Metagenomic Assembly Using Simulated Next Generation Sequencing Data. *PLOS ONE* 2012;**7**:e31386. https://doi.org/10.1371/journal.pone.0031386

8. Aguirre de Cárcer D, Angly FE, Alcamí A. Evaluation of viral genome assembly and diversity estimation in deep metagenomes. *BMC Genomics* 2014;**15**:989. https://doi.org/10.1186/1471-2164-15-989
